# Effects of a Large Dose of Veratrum

**Published:** 1871-08

**Authors:** T. G. Williams

**Affiliations:** Watertown, Wis.


### EFFECTS OF A LARGE DOSE OF VERATRUM.

WATERTOWN, WIS., *May 15th, 1871.*

DEAR EXAMINER,—The following may be of interest: Dr. Harrison, age 60, good constitution, took by mistake over a teaspoonful fluid extract verat. vir. He intended taking some "cough balsam," but took instead a bottle of veratrum, and drank a mouthful from it. The article was officinal, and a good active preparation, being the same as I am using in the usual quantity. Had eaten his breakfast over an hour before.

Poison taken 9 A.M. *9.10.* Found him anxious; pulse 100, medium soft; had taken ipecac. ℈j.; ordered sul. zinc, gr. j., followed by as much warm water as he could drink. *9.15.* Free emesis produced by using feather; gave more warm water; pulse 88. *9.18.* Pulse 70, irregular and soft; extremities and face cold. *9.20.* Pulse 65; gave hypodermic injection, sul. mor., gr. ⅓. *9.25.* Pulse 78, steadier. *9.30.* Pulse 90, soft; complained of feeling weak; warmed considerably; countenance became flushed. *9.40.* Pulse 115. *9.50.* Pulse 95, more irregular; gave freely strong coffee; strengthened heart action; during next two hours the pulse ranged between 105

and 88, not very steady. *12.* Had a free, spontaneous emesis; pulse 90; perspired freely; breathing good most of the time; felt comfortable; pulse steadily diminished in frequency, but gained in steadiness till 3 P.M., when it reached 60. Kept between 60 and 65 for the following twelve hours, after which it steadily gained during 24 hours, till it had reached the normal frequency, 78.

Have seen more alarming symptoms after small doses than followed in his case.

During the first twenty-five minutes, symptoms were grave; afterwards nothing severe occurred.

It seemed to me that the inj. morph. controlled the symptoms in five minutes, and kept up its effect till the last.

I regard strong coffee as the best cardiac stimulant we have. Could notice its beneficial effects very plainly in this case.

He has had facial erysipelas two or three times. To-day, four days after his accident, he has it developing again. Whether it has been developed by the veratrum, or a mere coincidence, I cannot tell. Reason for reporting the case is to show that large doses of so active a poison as veratrum may sometimes be taken with no serious results.

T. G. Williams.

